# Signatures of Depression in Non-Stationary Biometric Time Series

**DOI:** 10.1155/2009/989824

**Published:** 2009-06-24

**Authors:** Milka Culic, Biljana Gjoneska, Hiie Hinrikus, Magnus Jändel, Wlodzimierz Klonowski, Hans Liljenström, Nada Pop-Jordanova, Dan Psatta, Dietrich von Rosen, Björn Wahlund

**Affiliations:** ^1^Department of Neurobiology Institute for Biological Research, University of Belgrade, 11000 Belgrade, Serbia; ^2^Division of Bioinformatics, Macedonian Academy of Sciences and Arts, 1000 Skopje, Macedonia; ^3^Department of Biomedical Engineering, Technomedicum of the Tallinn University of Technology, 19086 Tallinn, Estonia; ^4^The Swedish Defence Research Agency, SE-16490 Stockholm, Sweden; ^5^Lab of Biosignal Analysis Fundamentals, Institute of Biocybernetics and Biomedical Engineering, Polish Academy of Sciences, 00901 Warsaw, Poland; ^6^Department of Energy and Technology, Swedish University of Agricultural Sciences, SE-75007 Uppsala, Sweden; ^7^Pediatric Clinic, Faculty of Medicine, University of Skopje, 1000 Skopje, Macedonia; ^8^Institute of Neurology and Psychiatry in Bucharest, 75622 Bucharest, Romania; ^9^Department of Clinical Neuroscience, Division of Psychiatry, Karolinska Instititute, SE-17177 Stockholm, Sweden

## Abstract

This paper is based on a discussion that was held during a special session on models of mental disorders, at the NeuroMath meeting in Stockholm, Sweden, in September 2008. At this occasion, scientists from different countries and different fields of research presented their research and discussed open questions with regard to analyses and models of mental disorders, in particular depression. The content of this paper emerged from these discussions and in the presentation we briefly link biomarkers (hormones), bio-signals (EEG) and biomaps (brain-maps via EEG) to depression and its treatments, via linear statistical models as well as nonlinear dynamic models. Some examples involving EEG-data are presented.

## 1. Introduction

When studying mental disorders researchers have primarily focused on gathering data, that is, the approach is basically empirical. In recent years, this has often been performed with advanced technical equipment. On the other hand, analysis of data often consists of rather elementary statistics, where comparisons are performed through testing hypotheses. There is also a growing literature on dynamics and non-linear modelling, in particular for EEG data, but very rarely it is distinguished *between* individual and *within* individual variation. To some extent, technology is far ahead of analytic tools and explanatory theories. The research mainly relies on the following well known paradigm: (i) make a model of the phenomenon under study; (ii) collect data by an experiment or sample survey; (iii) test the model using data; (iv) refine the model and restart. The weak point is of course the knowledge about the model. Einstein once rather drastically pointed out: “A theory can be proved by an experiment but no path leads from the experiment to the birth of a theory.” 

This paper is unique in so far that many researcher from many disciplines have met and discussed depression from different perspectives. The paper delivers a lot of bricks, but no house is built. In fact, an architect is missing. The aim of the paper is to share our experiences of a multidisciplinary view where hopefully empirically oriented researchers, as well as those who are more deductive can find new perspectives in modelling depression. Indeed, the project is a novel approach to help to uncover a serious mental decease, which would be hard to carry out within commonly structured academic institutions.

**Problems in Classifying and Modelling Major Depression**

The use of the current classification schemas, including DSM-IV, undoubtedly contributes to the difficulties in finding genes and biological variables for psychiatric disorders. They are based on clusters of symptoms and characteristics of clinical courses that do not necessarily describe homogenous disorders, and rather reflect common final pathways of different pathophysiological processes [1].

Moreover, biological variables and behaviours may not be associated on a simplistic, one-to-one basis; the true relationship between, for example, a gene and a behaviour, is probably more akin to the sensitive dependence on initial conditions in chaos theory. For example, there is presumably no gene for 'language'. Instead, there is a number of genes that pattern the embryonic brain in such a way as to facilitate and allow the physiological processes necessary for language acquisition. In a similar manner, no gene has been found to singularly code for a human psychiatric condition. To understand the pathogenesis and neurobiology of depression multidisciplinary research is necessary. 

A strategy to overcome the methodological difficulties mentioned above is the proposal of putative endophenotypes. The term “endophenotype” was described as an internal phenotype (i.e., not obvious to the unaided eye) that fills the gap between available descriptors and between the gene and the elusive disease process [2], and therefore may help to resolve questions about etiological models. Modelling Major Depression must be based on the state of art of knowledge of which psychopathological characteristics that are biologically and clinically meaningful and can be assessed quantitatively.

The endophenotypes may be defined at two levels, (a) the key components of major depression, that is, kern symptoms and stress sensitivity and (b) biological endophenotypes Not surprisingly, studies on the biological basis of depression have found stronger associations between specific biological dysfunctions and certain components of major depression symptoms, such as cognitive deficits, rumination, psychomotor retardation, anhedonia, and lowered mood have been associated with specific focal abnormalities of regional cerebral blood flow (CBF; [3, 4]). Thus, biological variables strongly related to key components are defined as biological endophenotypes.

*The key components of major depression are: (1) Depressed Mood (Mood Bias Toward Negative Emotions), (2) Anhedonia (Impaired Reward Function), (3) Impaired Learning and Memory, (4) Neurovegetative Signs, (5) Diurnal Variation, (6) Impaired Executive Cognitive Function (Response Speed), (7) Psychomotor Change (Retardation, Agitation), (8) Increased Stress Sensitivity (Gender Specific).*

*The biological endophenotypes are: (1) REM Sleep, (2) Abnormalities in Brain Structure and Function (Functional imaging, Structural imaging, Receptor pharmacology, Serotonin, Dopamine, and Norepinephrine), (3) HPA Axis and CRH, (4) Intracellular Signalling Molecules (Neurotrophic factors, Ubiquitous signalling cascades).*

In this paper we describe some potential biological endophenotypes, such as circadian rhythms, EEG findings and animal models. It is obvious that there is no simple model for depression, rather many complex models based on various scales from a micro-, meso- and macro perspective.

Finding appropriate models for mental disorders is the ultimate goal and we believe that for this purpose relevant inter-/multi-disciplinary knowledge is necessary. In this paper, we will briefly mention some advanced linear statistical models, which are useful when studying circadian rhythms (Sections 2 and 3), some ideas about complexity in signals (EEG data, Sections 4 and 5), and we also consider animal models (Section 7), which are important in generating models for humans. Moreover, four different examples which comprise EEG data are presented (Sections 6, 8, and 9).

## 2. Circadian Rhythms, Melatonin, and Bright Light Therapy

Circadian rhythms control, among other things, appetite, energy, mood and sleep. The study of these rhythms dates back to the 19th century. From about 1980 one began to study changes in physical strength, aerobic capacity, blood pressure, mental alertness, and secretion of neurotransmitters and hormones. Depression was then studied in relation to the disruption of biological clocks. In Seasonal Affective Disorder (SAD) the mood is closely connected with circadian rhythm disorder. Moreover, it seems that the Suprachiasmatic Nucleus (SCN), which may be viewed as a master clock of the body, has difficulties to follow the changes in the day and night cycle. A support for this hypothesis is the production of the hormone melatonin and its relation so SAD. Melatonin is produced by pinealocytes in the pineal gland, which is under the influence of SCN and is suppressed by daylight.

More than 20 years ago, depression was studied in relation to various hormones, in particular melatonin. Melatonin peak level was found lowered in acutely ill depressed patients, who also had hypercortisolemia and an abnormal dexamethasone suppression test in comparison to healthy subjects. The melatonin peak levels remained low when these patients were re-examined during remission, whereas the changes in the hypothalamatic-pictiutary-adrent-cortex axis disappeared (see Wetterberg et al. [5], Beck-Friis et al. [6]). Therefore, melatonin levels may be viewed as biomarkers for depression.

During the 1980s, Bright Light Therapy (BLT) was introduced with a clear therapeutic effect on approximately 85% of the patients with SAD, as well as on patients with bipolar disorder. Most studies support that BLT normalizes circadian rhythms, that is, the phase and the melatonin level (amplitude) may vary between individuals, as well as states of physiological conditions and disorders (cf. [7, 8]). 

It is important to have appropriate designs of experiments, strong statistical/mathematical tools, and specialized knowledge concerning the hormones under investigation. For example, the effect of age, gender, body weight/height on the melatonin level is important to take into account. Initially, circadian melatonin rhythms were analysed with ordinary regression analysis and trigonometric functions. Studies were often designed so that around 10 serum measurements per individual were taken over the day and night cycle. Usually between 10 and 50 individuals from different diagnostic groups, including a control group, were studied with an aim to compare the treatment groups with respect to the hormone profile over the day and night cycle. The main problem is that melatonin is not stable in serum, since it is rhythmically released within relatively short time intervals. With a sampling strategy of about 10 measurements over 24 hours the release of melatonin is impossible to capture and the variation will be built in as a measurement error.

## 3. Statistical Repeated Measurements Models for the Analysis of Circadian Rhythms: An Example of Linear Models Analysis

Since there are often repeated measurements on individuals in depression studies one has to apply some of the repeated measurements tools that nowadays are available, and not use standard regression methods. The difficulty lays in finding an appropriate model for the covariance structure within individuals. A conservative approach is to assume an arbitrary covariance matrix and if melatonin sampling has taken place at the same time points for all individuals we may apply the classical Growth Curve model due to Potthoff and Roy [9] or generalized Growth Curve models (cf. Kollo and von Rosen [10, Chapter 4]). Otherwise, we may rely on mixed linear models analysis (cf. Fitzmaurice et al. [11]) with random parameters, which is suitable for analyzing short time series. However, this approach usually gives only asymptotic correct results.

Nowadays, for example, melatonin is often sampled via an inserted indwelling intravenous catheter and samples are collected every, say, 15 minutes. This calls for more advanced methods than the suggested analyses above, which are extensions of classical multivariate variance analysis. New methods have to be developed in order to estimate melatonin profiles and to perform rigorous significance tests. Of course, one can always create summary statistics, but it is challenging to make use of the full sampling resolution. In the future, we will probably see more of high-dimensional statistical analysis or stochastic process approaches.

## 4. Techniques for Identifying Depression from EEG

An even more advanced method than to model high-resolution hormone samples is to study bio-signals, such as EEG and sometimes EEG in addition to hormones, in particular when assessing BLT. The information from EEG is of a completely different type than, for example, serum melatonin concentration, and it is important to study these often nonstationary time series.

As behavioural alterations are based on neurophysiology, behaviour should be studied in association with brain activity correlates—single neuronal discharges, local field potentials and EEG/ECoG. In other words, the behavioural changes in food intake, sleep patterns, work habits and general motor activity are quite conspicuous in depression, in humans as well as in animals, and may be quantified with changes in EEG. Moreover, antidepressant treatments (drug therapy, moderate physical exercise, electroconvulsive shock) reverse more or less the EEG changes found in human depression, or in certain animal models of depression. In this way, brain lateralization effects of depression are electrophysiologically evident. The brain-rate parameter may serve as an effective integral indicator of these changes (Pop-Jordanova and Pop-Jordanov, [12]). Neuronal models and other approaches for electroencephalographically identifying/quantifying depression may act complementarily. In order to refine the treatment procedure, we may suggest more specified electrocortical stimulations in an animal model, and various analyses in complexity of acquired electrical brain signals by multichannel chronic recording techniques with telemetry technology (Culic, [13]).

## 5. EEG Signals and Complexity Measures: An Example of Non-Linear Analysis

In EEG signals, the classical statistical approach of interpreting measurement errors as generators of uncertainty is not valid. For EEG, most of the noise seems due to model error, which cannot be considered to be random. We have not seen any EEG model where residuals are completely randomly distributed around a fitted model. This may be due to dependence structures but it is not clear how to estimate this dependency in non-stationary series. Therefore, it is reasonable that when studying EEG completely different tools than classical statistical ones are advantageous. For example, non-linear dynamic models may play an important role. Moreover, the number of used electrodes is important. With many electrodes we can analyze spatio-temporal models, that is, brain-maps. Below, a non-linear method of complexity analysis is presented. For references to other non-linear methods for studying complex EEG signals, see Freeman [14] and Perlovsky and Kozma, [15].

Methods that assess signal complexity, like fractal and symbolic methods, may be suitable. For example EEG-signal complexity measured by Higuchi's fractal dimension in time domain, *D*_*f*_(*t*), is proposed in assessing BLT for treatment of SAD (cf. Klonowski [16]). *D*_*f*_(*t*) is calculated for EEG-signal on each electrode on the scalp and then a spatio-temporal map of complexity measured by fractal dimension can be made. The term ‘fractal dimension’ may have different meanings, so it is necessary to emphasize that *D*_*f*_(*t*) measures local complexity of the curve representing the given signal—a simple curve has always a dimension equal to 1, while a plane has a dimension equal to 2, so local complexity of a curve on a plane may be characterized by a number between 1 and 2, with 2 corresponding to pure noise (the curve “filling up” the whole plane). For assessing signal complexity using Higuchi's *D*_*f*_ in time domain it is not important if the signal is “really” chaotic (which makes the curve that represents the signal showing fractal properties), and neither deterministic nor random.

## 6. Example 1: An Example Where Complexity is Studied

To illustrate a non-linear analysis technique, the EEG from 10 patients suffering of SAD and treated with BLT, was analyzed. The data were collected before and 2 weeks after BLT was applied. It was demonstrated that in patients suffering of SAD, the mean D_f_ of the EEG-signal was smaller than in healthy subjects—BLT increased the mean value of D_f_ in those suffering of SAD (cf. Klonowski et al. [17]). For every patient epochs with a duration of approximate 20 seconds length, starting about 5 seconds before eyes-opening and ending about 5 seconds after eyes-closing were analyzed. When an eyes-opening event occurred, the fractal dimension of the EEG-signal (based on windows of 100 observations) increased from 1.1–1.3 to 1.5-1.6 in the occipital channels and even to 1.8 in the frontal channels. This increase is denoted by Δ_o_. When the eyes remained open, the fractal dimension diminished, and rose again when an eyes-closing event occurred; when the eyes remained closed, it diminished again. This decrease is denoted by Δ_c_.

The open-/closed-eyes fractal dimension ratio (FD-ratio), that is, Δ_o_/Δ_c_ was investigated. For a clinical assessment of the patients the Hamilton Depression Rating Scale (HDRS), [18] was used It was observed that in EEG of healthy subjects the * FD-ratio* was close to 1, while for patients with high HDRS the *FD*-ratio differed from 1.0. For SAD patients the *FD-ratio* was compared with HDRS before and after BLT. For those patients for whom HDRS diminished after BLT the *FD*-ratio “normalized”—it became closer to 1.0. 

In the material mentioned above, there was a focus on possible BLT effects on the EEG. One of the main problems of studying depression is that patient groups are very inhomogeneous with a strong individual component of response. Besides this, there are covariables such as age, gender and body height/weight which influence the measured response. Moreover, patients may have used medicines which even after wash-out periods may have an effect on the results.

As a complement, one can consider ADHD children. They constitute an interesting group, since they usually have not undergone pharmacological treatment, and therefore could be used as a control group (Zorcec et al. [19]). However, in order to propose initial theories, the best is probably to start with animal models.

## 7. Animal Models for Developing Antidepressive Treatment: A Short Introduction

The overall goal is to understand the interplay between structural, chemical, and electrical signals in the brain, which gives rise to a depressed behaviour in humans. In particular, studies of animal models of depression may be important for performing screening tests to discover and develop new antidepressant drugs. Moreover, animal models are used to simulate and elucidate neurobiological aspects of depressive illness—to induce anhedonia as a core symptom of depression, or to a particular subtype of depression, and to examine mechanisms of depressive syndromes and of various acute and chronic antidepressive treatments (Mitchell and Redfern [20]; Harro [21]; Sarbadhikari and Sankar [22]; Willner [23, 24]; Porsolt et al. [25]). For instance, disruption of neurochemistry of the noradrenergic *locus coeruleus* (LC) is at least one aspect of the pathophysiology of major depression (Klimek et al. [26]). The mutual role of LC and cerebellum is also bringing new information about motor and non-motor cerebellar processing (Culic et al. [27]). The fitness of an animal model depends on the similarity with the human disorder with respect to symptomatology, etiology, biochemistry, electrophysiology and response to antidepressive treatment. It is of vital importance to fully recognize the limitations of such models.

Certain behavioural or physiological responses, which are supposed to be important for depression, are measured in animal assay models. Examples of assay models are: muricide, potentiation of yohimbine lethality or amphetamine-induced hyperactivity, antagonism of apomorphine-induced hypothermia, preferential reduction of kindled seizures initiated from the amygdale, and facilitation of circadian rhythm readjustment. Such models focus on the predictive value for screening of new drugs and other treatments, without trying to create a human disorder in the animal. On the other hand, homologous animal models place less emphasis on correlative approaches and rely more on construct and face validity. They are based on resemblance to symptoms of human depression although some symptoms can never be mimicked in animals. Homologous examples include: forced swim test, tail suspension test, electrolytic lesioning of the dorsomedial amygdala, exhaustion stress, and chronic mild/variable stress-induced anhedonia.

## 8. Example 2: An Example with Experimental Neurosis in Cats

That EEG correlates of motivation and short term memory (STM) in cats during an approach-avoidance delayed differentiation task were studied several decades ago (Psatta [28, 29]). The preparatory (cue) stimuli were tone and intermittent light stimulation (ILS), the delay of ten seconds, and reinforcement was either food or pain. Under these conditions, motivation changed from one trial to another. EEG activity varying with motivation during the delayed period was statistically confirmed. Changes occurred only in the amygdaloidal and the hypothalamic nuclei. Changes significantly related to STM (either prolonged desynchronization or ILS memory traces during the delay period) occurred only in the cortical cognitive areas (Ectosylvius medius or Marginalis posterior in cats). The electrical activity in the Hippocampus had complex relationships. A prolonged theta activity in the Dorsal Hippocampus (DH) accompanied by fast activity in the Ventral Hippocampus (VH) occurred when STM was successful, attention sustained and the motor response delayed. Occurrence of theta activity in VH systematically accompanied the motor response. It was concluded that hippocampus exerts a complex sensory-motor integration. DH intervenes in the sensory processing of information, by closing the thalamic gates. Any fast activity (even short) in DH was accompanied by the loss of STM correlates (weak, fast stimulations of DH also blocked STM). VH exerts an inhibitory motor control on the frontal cortex (by uncial fibers).

Depression occurred in five out of 13 investigated animals, when the approach-avoidance conflict induced manifestations of experimental neurosis (Psatta [30, 31]). The most characteristic electrical change in these animals was the constant occurrence of a mid-amplitude very fast rhythm in both DH and VH. Thus depression was attributed to the exaggerated inhibitory control of hippocampus induced by the emotional conflict imposed. The experiment permitted an exploration of the adrenergic/cholinergic ratio contribution to the deviated behaviour. Administration of small doses of Reserpine, reducing the cerebral amount of free catecholamines, induced a reoccurrence of the theta rhythm in the hippocampus and of the coordinated motor responses. Further administration resulted in change in the appearance of higher amplitude fast rhythms in the hippocampus and of exhaustion type of depression.

It was concluded that there is an optimal monoamine level (a narrow window) for which hippocampus acts normally. Administration of Nialamide after Reserpine restored first theta activity and eventually the original aspect of mid amplitude fast rhythms in the hippocampus (the anxious type of depression). A choline-estherase inhibitor (eserine) in small doses restored in the depressed animals both the DH theta activity and the EEG correlates of STM (the memory traces). In higher doses, Eserine induced instead an extreme agitation of those animals (on a high level cholinergic/adrenergic equilibrium). Atropine after Reserpine induced a release of motor behaviour, but no signs of performing STM. These experiments reveal the difficulty of controlling depression by psychotropic drugs administration. It is also outlined the difficult extrapolation of these results in animals to experimentation in humans in whom subcortical EEG investigation is not possible. 

In order to replicate the described experiment, the authors tried the effects of Go-NoGo STM performances in humans (using auditory click cue stimuli and slightly delayed motor finger responses). They considered that the rolandic reaction evidenced by EEG Spectral Reaction Mapping was the equivalent of the cognitive cortical areas response encountered in animals, whereas the N220/P300 components of the Auditory Reponses evoked by the click stimuli, are the equivalent of the fast and slow rhytms occuring in the Hippocampus in animals. N220 is larger in NoGo, P300 is deeper in Go situations. Both these EEG manifestations disappear in case of Temporal Lobe Epilepsy (and Neurosis), and are enhanced in case of Frontal Lobe Epilepsy (Psatta and Matei, [32, 33]).

## 9. Example 3: Exposure Experiments

Finally, we mention two different exposure treatment/experiments which also indicate that EEG is appropriate to study when investigating mental disorders.

Electroconvulsive therapy (ECT) is a treatment in which seizures are electrically induced in anesthetized patients for therapeutic purposes. ECT is most often used as a treatment for severe major depression, where patients have not responded to other treatments. Seizures may be monitored by EEG, electrocardiogram (ECG) and electromyogram (EMG). The course can be summarized as three events of distinct and sequential phase patterns. The first event contains high-voltage “sharp waves and spikes,” the second rhythmical “slow-waves” and the third event an abrupt and well-defined ending.

Most ECT studies have investigated the physiological mechanism of action in relation to clinical response [34]. A typical study runs as follows: subjects with unilateral electrode placement according to the d’Elia method [35] receive bi-directional pulse ECT; ECT is routinely administrated three times a week for a period of 2–4 weeks; each time EEG is recorded it covers the above mentioned three distinct phases. Studies consist often of about 30 patients who are followed 5–9 times. This results in a huge data set with a possibility to test several interesting hypothesis, for example “can one subgroup depressive disorder with the aid of seizure data?” Moreover, one problem with ECT is that it is not known what happens in the brain during or after treatment. In order to test various hypotheses of ECT effects (e.g., on neural network connectivity by stimulation of nerve sprouting or nerve deletion), computational models of cortical neural networks may be useful (Gu et al. [36–38]). 

Microwave exposure is a method in which the electromagnetic radiation at field power densities is much lower than used in ECT is applied for treatment of mental disorders. For example, mood improvement in patients with bipolar depressive disorder has been reported at the field power density within clinical magnetic resonance system limits [39]. Exposure to 450 MHz microwave radiation modulated at 1000 Hz frequency at the field power density 0.9 mW/cm^2^ has been shown to cause short-term alteration in mood of major depressive disorder [40]. The experiments were carried out on a group of depressive patients (18 females) and a control group of healthy volunteers (18 females) exposed by microwave radiation during 30 minutes. Subjects with nonpsychotic major depressive disorder are defined by ICD-10 criteria and determined by the 17-item HDRS score (as in Example 1). The average HDRS score for the group was 21 (s.d. 3.3). All the subjects passed two experimental procedures—with exposure and sham. As a subjective criteria of microwave effect, the Brief Affect Scale (BAS) and Visual Analogue Scale (VAS) before and after each exposure and sham procedure were used. The resting 9 channel EEG was recorded during the experiment.

As a measure for evaluation of the mood improvement the spectral asymmetry index (SASI) as a combination of the EEG beta and theta power was selected [41]. The BAS revealed a minor improvement (11 subjects) in subjective mood score after exposure and VAS test revealed significant change between scores before (average 33.3) and after (average 40.2) treatment for exposed subjects and no significant change for sham exposed subjects. 

The EEG analysis detected objective effects of the treatment. The calculated SASI values were positive for depressive and negative for healthy subjects. Correlation between HDRS score and SASI values was 0.67. Exposure to microwave during 30 minutes reduced SASI values for depressive patients: average SASI value was 0.16 for exposed and 0.19 for sham exposed recordings. The analysis revealed statistically significant differences between exposed and sham exposed patients. These preliminary results are promising and the SASI method of EEG analysis for mood evaluation as well as microwave exposure for treatment of mental disorders need further investigations. Variations between individuals and within individuals should be investigated and experiments on different groups should be performed.

## 10. Concluding Remarks

In this paper we have focused on the neural understanding of depression. The aim is to find a link between physiology and mental disorders. There are several indications for such links, for example between hormone levels and SAD. Also EEG analysis reveals connections with SAD. In the future, we hope to have found models that would manifest connections between depression and bio-markers, bio-signals, and bio-maps, such as hormone levels, EEG, fMRI, and so forth. To achieve this goal statistical/mathematical theory has to be developed together with experimental designs. In particular, we have to learn how to take into account *between* and *within* subject variations in spatio-temporal, parametric or semi-parametric models.
